# Noninvasive Ventilation for Preterm Twin Neonates with Respiratory Distress Syndrome: A Randomized Controlled Trial

**DOI:** 10.1038/srep14483

**Published:** 2015-09-24

**Authors:** Long Chen, Li Wang, Jie Li, Nan Wang, Yuan Shi

**Affiliations:** 1 Department of Pediatrics, Daping Hospital, Research Institute of Surgery, Third Military Medical University, Chongqing, 400042, China; 2 Department of Obstetrics and Gynecology, The First Affiliated Hospital of Chongqing Medical University, Chongqing, 400014, China

## Abstract

Noninvasive ventilation has been proven to be effective strategies for reducing the need for endotracheal ventilation in preterm infant with respiratory distress syndrome (RDS), however the best option needs to be further determined. A single center, paired design, randomized, controlled trial was conducted between Jan 2011 and July 2014. Preterm twins with RDS were included. One of a pair was randomized to NIPPV, while another to NCPAP. Surfactant was administrated as rescue treatment. The primary outcome was the need for endotracheal ventilation. The secondary outcomes were the complications. 143 pairs were randomized and 129 pairs finished the trial. The rates of endotracheal ventilation did not differ significantly between NIPPV and NCPAP groups (11.9% vs 19.6%, *P* = 0.080). This difference was not observed in the subgroup of infants who received surfactant therapy (11.1% vs 19.7%, *P* = 0.087). No secondary outcomes also differed significantly between the two groups. NIPPV did not result in a significantly lower incidence of intubation as compared with NCPAP in preterm twins with RDS.

In preterm infant with respiratory distress syndrome (RDS), endotracheal ventilation and exogenous surfactant replacement therapy are two important cornerstones to reduce neonatal mortality^1^. Although improving the survival^2^, endotracheal ventilation is actually related to increasing risks of infection and ventilation-associated lung injury. Importantly, prolonged duration of endotracheal ventilation induces more death, neurologic impairment, and bronchopulmonary dysplasia (BPD) in the post-neonatal period^3^. These complications and sequelea induce increased medical burden^4^. How to reduce the need for endotracheal ventilation and subsequent complications constitutes a challenge for neonatologists^5^.

To this day, early use of noninvasive respiratory support strategies has been suggested to be the most effective pathway to reduce those risks. Nasal continuous positive airway pressure (NCPAP) and nasal intermittent positive pressure ventilation (NIPPV) are two widely used ways of noninvasive ventilation strategies in preterm infant. As compared with invasive ventilation, NCPAP reduces the risks of BPD and abnormal neurodevelopment. However, there is only 60% success rate of avoiding intubation in the preterm neonate supported with NCPAP^6^. Supplying with an intermittent peak pressure on NCPAP, NIPPV is considered as a strengthened version of NCPAP with increased flow delivery in the upper airway, increased minute volume and functional residual capacity and recruitment of collapsed alveoli, improved stability of the chest wall and reduced asynchrony of thoraco-abdominal movement^7^,^8^,^9^, which have been proven to be crucial to decrease the incidences of invasive ventilation, BPD and death^10^. Several studies have compared NIPPV with NCPAP in non-twin neonates with RDS, which were mainly conducted in very preterm infants (less than 32 weeks’ gestational age). There was a rarity of similar study in moderate and late preterm infants (between 32 and 36 weeks’ gestational age)^11^. Actually, moderate and late infants accounted for more than 80% of preterm births^12^,^13^. Studies are needed to determine the impact of respiratory distress coupled with mild-moderate prematurity on short-term and long-term outcomes^14^.

The purpose of the present study was to compare NIPPV with NCPAP on the need for endotracheal ventilation and subsequent complications in preterm twins, especially in moderate and late preterm twins.

## Results

### Study neonates

**Figure 1:**
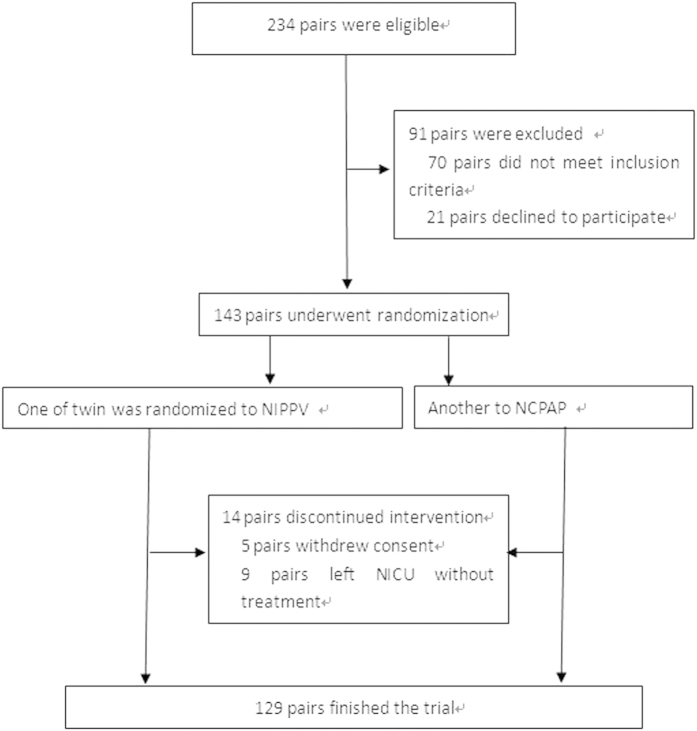
The flow diagram.

234 pairs were screened, of which 143 pairs underwent randomization and 14 parents of twins did not continue the interventions in the follow-up, and 129 pairs were ultimately enrolled and finished the trials, (129 in NIPPV group with 60 first born and 69 second born; 129 in NCPAP group with 69 first born and 60 second born) (Fig. 1). All preterm twins undergoing randomization, including those who lose to follow-up, were included in the final analysis. The number of involved twins reached the calculated needed sample size. Analysis according to the intention to treat principle was considered to be the most proper way of analyzing randomized controlled trial results. Data analyses followed the intention to treat principle. Intention to treat principle analysis required all randomized participants to be included and analyzed according to the treatment group to which they were originally assigned^15^.

All the neonates arrived at the NICU within six hours. There were also no significant differences in main clinical characteristics of neonates at birth weight, the ratio of the first or second born twin, Apgar score, and gender between the two groups (Table 1). Among them, 47(32.9%) pairs come from an egg. Antenatal steroids were given to 135(94.4%) pregnant women. Preterm premature rupture of the membrane, pregnant diabetes, hypertensive disorders and intrahepatic cholestasis of pregnancy appeared in 94(65.7%), 40(28.0%), 46(32.2%) and 39(27.3%) pregnant mothers, respectively. 89(62.2%) women received urgent cesarean delivery, 22(17.1%) in vaginal delivery, and the others (20.7%) received selective cesarean delivery.

### Primary and secondary outcomes

Except for the intervention, the twins received the same clinical treatment including surfactant administration. 122 pairs received endotracheal administration of surfactant within six hours after admission, and other parents of 21 pairs rejected surfactant administration. There was no significant difference in rate of intubation (11.9% vs 19.6%, *P* = 0.080) between the two groups. This difference was also not observed in the subgroup of infants who received surfactant therapy (11.1% vs 19.7%, *P* = 0.087). (Table 2). No secondary outcomes differed significantly between the two groups (Table 3). Other than, no gastrointestinal perforation/ dilatation and nasal trauma were observed.

### Subgroup Analyses

In subgroup analyses, when surfactant was administrated to rescue infants, those infants whose gestational age between 32–33 weeks were showed statistically significant difference between NIPPV and NCPAP groups (13.7% vs 31.4%, *P* = 0.049) (Table 2). However, the 95% confidence interval covered 1 (0.350–9.138). To further assess the effects between treatment and gestational age on the rate of intubation, the test of treatment-by-gestational age subgroup interaction was also conducted, and no interaction was observed (χ2 = 0.234, *P* = 0.628).

## Discussion

In this single-center, paired design, randomized, controlled trial, we aimed to decrease the incidence of endotracheal intubation in preterm twins with RDS, especially in moderate and late twins, through comparing NIPPV with NCPAP. As a result, we found that NIPPV did not reduce significantly the need for endotracheal ventilation as compared with NCPAP (11.9% vs 19.6%, *P* = 0.080). Similarities also appeared in the subgroup of infants who received surfactant therapy (11.1% vs 19.7%, *P* = 0.087) and the secondary outcomes.

In the past, several studies have compared the effects between NIPPV and NCPAP on the incidence of intubation, and the results were inconsistent. Meneses J *et al.*^16^ indicated that NIPPV did not significantly reduce the intubation rate in the first 72 hours comparing with NCPAP, of which mean gestational ages were about 30 weeks. The largest multi-centered study on the comparison of NIPPV and NCPAP also showed that, in very preterm infant, there was no significant difference on the rate of intubation and survival to 36 weeks of post-menstrual age without BPD^17^. In 2014, Kugelman A *et al*. reported a randomized pilot study comparing heated humidified high-flow nasal cannulae with NIPPV for RDS, which showed similar effect on preventing endotracheal ventilation in premature infants suffering from RDS with mean gestational age of 32 weeks^18^. In the present study, our data did not also showed difference in intubation rates, which was consistent with the newly up-to-date meta-analysis of Wang L *et al.*^19^ In contrast, a few previous meta-analyses^20^,^21^,^22^ gave preference to the use of NIPPV. Our previous study including both preterm and term infants with RDS also suggested that NIPPV might have better effects as compared with NCPAP^23^.

One cause to explain the inconsistence among different studies might be gestational age. Previous studies were mainly enforced in very preterm infants. Nowadays, newborn infants were actually divided into post-term (>42 weeks), full term (39–41 weeks), early term (37–38 weeks), late preterm (34–36 weeks), moderate preterm (32–33 weeks), and very preterm (<32 weeks). In the very preterm infants, the incidence rate of RDS gradually has been confirmed to be increased with decreasing gestational age. EuroNeoStat figures for 2006 showed an incidence of 92% at 24–25 weeks’, 88% at 26–27 weeks’, 76% at 28–29 weeks’, and 57% at 30–31 weeks at gestational age^24^. In the infants with gestational age less than 30 weeks, an obviously increase was observed in the incidence rate of RDS. It might therefore be improper to conduct the trials in very preterm infants with long time span, and the gestational age below 32 weeks should be divided into more subgroups, such as 30–32 weeks, 28–32 weeks and 26–28 weeks and so on. Similarities also appeared in other stages of the preterm. In the present study, three subgroups related to gestational age were divided and the results did not reveal difference between NIPPV and NCPAP groups.

Another important cause might be the baseline differences of pregnant women. As is known, several diseases of pregnant women can obviously influence the severities of RDS^25^. Different levels of gestational diabetes mellitus, administration of dexamethasone, premature of rupture of fetal membranes, and intrahepatic cholestasis of pregnancy can affect the lung maturation of newborn^26^. Previous studies only showed simple homogeneities of “absent” or “present” in pregnancy-associated diseases of mother between the two groups, and these homogeneities might be not true conditions of homogeneity. In the present paired design study, these differences could be the best homogeneity in preterm twins, thus, the results should be the higher reliability. Our results were consistent with the studies of Kirpalani, H. *et al.*^17^ and Kugelman, A. *et al.*^18^. However, the sample sizes were very different among the three studies (258 vs 1009 vs 76). It was easy to understand the better homogeneities of pregnant women with larger sample size. As far as the smaller sample size was concerned in the study of Kugelman, A. *et al*., a reasonable speculation was that the study might show the similar homogeneity with us.

The last but not least, the cause might be surfactant administration. Generally, RDS may gradually aggravate in 72 hours with the consumption of replacement surfactant. Therefore, prophylactic, early and enough surfactant replacement therapy can effectively reduce the incidence of intubation and complications in preterm infants with RDS as compared with later selective surfactant administration^27^, which was consistent with the report by Duman N *et al.*^28^ The updated meta-analysis showed that, compared with NCPAP group, there was a significant decrease in the need for invasive ventilation in NIPPV group in the preterm infants who received surfactant^19^. In the present study, surfactant administration was enforced in 85.3% (122/143) twin neonates with mean 1.5–1.6 times in early stage, and the overall rates of intubation were similar between the two groups. Although in subgroup analysis of 32–33 weeks’ gestational age, statistically significant lower rate of intubation was found in NIPPV group as compared with that in NCPAP group, the 95% confidence interval covering 1. Our result was inconsistent with the updated meta-analysis^19^.

In addition, the present study was only concerned with non-synchronized NIPPV. Several studies have reported the advantages of synchronized NIPPV. Compared to NCPAP, the use of synchronized NIPPV was related to reduced intubation rate^29^,^30^, thoracoabdominal motion asynchrony^31^, and the work of breathing^32^, as well as increased tidal volume and minute volume^33^. Gizzi C revealed that synchronized NIPPV seemed more effective in reducing the incidence of desaturations, bradycardias and central apnoea episodes in preterm infants^34^. Nevertheless, retrospective data indicated that use of synchronized NIPPV was associated with similar impact on clinical outcomes as compared with non-synchronized NIPPV^35^. Moreover, Owen LS *et al*. reported that NIPPV delivered fewer pressure peaks at lower pressures when the respiratory rate was >55/min^36^. Those trials were mainly conducted in very preterm infants. Further trials were needed in moderate and late preterm infants.

Only few studies reported the risks of noninvasive ventilation, such as gastrointestinal perforation^37^ and nasal trauma^38^. And these studies were mainly performed in the pre-surfactanct era. Morley CJ *et al*. demonstrated more incidences of pneumothorax in CPAP group^39^, and the air leak was related to epinephrine and surfactant use, and prenatal steroids were protective factor^40^. Other than, Sai Sunil Kishore M *et al*. also reported a slight increase in abdominal distension in the NIPPV group. In the present study, we did not found any significant differences in related to complications between NIPPV and NCPAP groups, which was consistent with the meta-analyses^19^,^41^.

The major limitations of the study: 1) no preterm twins below 28 weeks’ gestational age; 2) relatively small sample size in the subgroups. They might induce potential bias, including restricted application scope and size effect. These problems could be overcome in additional studies. Given the potential limitations, more trials are needed in the future.

In summary, among preterm twins with RDS, including the infants administrated with surfactant, NIPPV might be not prior to NCPAP with respect to avoiding intubation and reducing subsequent complications as the primary respiratory support in the early life.

## Methods

### Study Design and Participants

This was a single-center, paired design, randomized controlled trial conducted in a tertiary neonatal intensive care unit (NICU) from Jan 2011 to July 2014 at Daping Hospital, Third Military Medical University, China. The trial was approved by the ethics committee of the hospital and registered at http://www.clinicaltrials.gov. (ID: NCT01926106)(the registration date: 08/19/2013). It was from a prospective protocol. Informed parental written consents from all subjects were obtained. The trial was performed in accordance with the approved guidelines and regulations. All operations in the trial were performed in accordance with relevant guidelines and regulations

Included criteria: (1) the gestational age was from 28 to 36 weeks; (2) these infants were twins; (3) they were diagnosed as RDS. The diagnosis of RDS was based on clinical manifestations and chest X-ray findings, which was similar in twin neonates. The clinical signs and symptoms of RDS were respiratory distress, tachypnea, nasal flaring, groan, and cyanosis after birth. The typical X-ray picture of RDS showed a grain shadow, air bronchogram or white lung, and X-rays of twin neonates must be the same grades. X-rays were judged by two radiologists blinded to the patient’s condition. Infants were excluded from this study if they were not fit for the use of NIPPV or met any of the following criteria: different clinical manifestations and/or grades of radiological findings in twin neonates, major congenital anomalies, intubation at admission to NICU because of severe conditions such as clinically severe respiratory distress with severe respiratory acidosis (PaCO2 > 60 mmHg), neonatal pulmonary hemorrhage, cardiopulmonary arrest without effective resuscitation needing continued ventilation and rescue, and died or left the NICU within 24 hr and/or before randomization.

### Allocation and Blinding

A table of random numbers concealed in opaque envelops was used to allocate and blind. After documenting parental consent, one of an eligible twin was randomly allocated to NIPPV, while another to NCPAP. Blinding for doctor was not possible due to the nature of the intervention.

### Study Intervention

A time-cycled, pressure-limited and continuous-flow neonatal ventilator (Babylog 8000, Drager, Germany) was used for neonates assigned to the NIPPV group in a non-synchronized mode. The initial settings were: frequency of 10–30 breaths/min, peak inspiratory pressure (PIP) of 15–25 cm H_2_O, and positive end expiratory pressure (PEEP) of 4–6 cm H_2_O. The fraction of inspired oxygen (FiO_2_) was regulated from 0.21 to 0.40 in order to maintain oxygen saturation (SpO_2_) from 90% to 95% by a pulse oximeter. Neonates assigned to the NCPAP group were initiated on a pressure of 4–6 cm H_2_O by bubble CPAP system (Stephan), with FiO_2_ from 0.21 to 0.40 to maintain SpO_2_ from 90% to 95%. To avoid stomach/intestine dilatation, a tube was used from mouth to stomach when the interventions were conducted.

When the neonate was admitted to the NICU and had fulfilled the entry criteria, NIPPV or NCPAP was started immediately on the basis of the group assignment. Other care was at the discretion of the attending neonatologist. Pulmonary surfactant (Curosurf, Chiesi Pharmaceuticals, Parma, Italy) was administered with a dosage of 100 mg/ kg as a rescue treatment if an infant needed FiO_2_ > 0.40 to maintain the targeted SpO_2_. We used the INSURE (intubation-surfactant-extubation) technique of surfactant administration^42^

### Clinical Data

The clinical data of all enrolled neonates were recorded, including main clinical characteristics, intubation, surfactant administration and complications within 100 days. 100 days were defined for the following causes: 1). The gestational age of the smallest infant included was 200 days, and 100 days after birth ensured the corrected age to 40–44 weeks. 2). the observation time of the primary and secondary outcomes were 100 days. And 100 days later, the assessment of the primary and secondary outcomes were discontinued.

### Evaluation of Outcomes

The primary outcome of this study was to determine the need for endotracheal ventilation in twins randomized to the two groups. Neonates were intubated if they were not improved and needed mechanical ventilation, which was based on the standard indication^43^. The criteria for intubation and mechanical ventilation were as follows: severe respiratory acidosis (PaCO_2_ > 60 mmHg), severe apnea and bradycardia (defined as recurrent apnea with >3 episodes per hour associated with heart rate <100/min, a single episode of apnea that required bag and mask ventilation, or associated with hypoxemia with SpO_2_ < 85% and FiO_2_ > 0.6), severe respiratory distress, neonatal pulmonary hemorrhage, and cardiopulmonary arrest without effective resuscitation needing continued ventilation and rescue. The secondary outcomes included surfactant administration and the incidences of bronchopulmonary dysplasia (BPD), patent ductus arteriosus (PDA), retinopathy of prematurity (ROP), necrotizing enterocolitis (NEC), intraventricular hemorrhage (IVH) and possible side effects of the noninvasive modes. To further evaluate the effects of surfactant and gestational age on the rate of intubation, the subgroups were defined and compared in these infants administrated with surfactant. The defined subgroups of gestational age were as follows: 1) less than 32 weeks, 2) 32–33 weeks, 3) 34–36 weeks.

For all neonates, effective NIPPV was defined as avoiding intubation successfully, and ineffective NIPPV was defined as intubation within 100 days after birth. And similarity appeared in NCPAP.

### Sample Size Estimation

The sample size estimation was calculated by PASS software (2008 v 8.0.3). According to previous studies^16^,^44^,^45^, average 40% of preterm neonates administered with early NCPAP and surfactant treatment for RDS needed endotracheal ventilation. Our previous experience has indicated that the success rate of NIPPV and NCPAP was about 90% and 80%, respectively^45^. A plausible estimate of the coincidence rate both NIPPV and NCPAP success is 70%. With 80% power and a 2-sided significance level of 0.05, 114 neonates would be needed at least in each group.

Actually, during the study period from Jan 2011 to July 2014, 129 pairs were enrolled and finished the trials. The success rate of NIPPV and NCPAP were about 88.4% (114/129) and 79.8% (103/129), the coincidence rate both NIPPV and NCPAP success is 72.1% (93/129), with 80% power and a 2-sided significance level of 0.05, 123 neonates would be needed at least in each group. Therefore, the actual sample size was more than theoretical need.

### Statistical Analysis

Continuous variables, expressed as mean ± standard deviation, were compared using paired samples t test. Categorical variables were compared using the McNemar’s test. Predefined three subgroups were <32 weeks, 32–33 weeks and 34–36 weeks, and subgroup analyses were conducted for the primary outcome in the preterm infants administrated with surfactant. Other than, to further justify the effect of surfactant on intubation within subgroup, the test of treatment-by-gestational age subgroup interaction was also conducted using the paired binary logistic regression. For the preterm infant loss to follow-up, the missing values of the primary and secondary outcomes were replaced using multiple imputation. All analyses were carried out using computer software (SPSS 16.0 for windows). *P*-values less than 0.05 were regarded as statistically significant.

## Additional Information

**How to cite this article**: Chen, L. *et al.* Noninvasive Ventilation for Preterm Twin Neonates with Respiratory Distress Syndrome: A Randomized Controlled Trial. *Sci. Rep.* **5**, 14483; doi: 10.1038/srep14483 (2015).

## Figures and Tables

**Table 1:** Main clinical feature at birth.

Clinical data		nIPPV (143)	nCPAP (143)
Birth Age(days)		224.6 ± 10.7	
Weight(g)		1831.3 ± 258.6	1842.8 ± 292.3
the first born: the second born twin		67:76	76:67
Gender (male: female)		78:65	90:53
Apgar score	1 min	6.7 ± 1.3	7.0 ± 1.4
	5 min	8.6 ± 0.6	8.5 ± 0.6
	10 min	8.9 ± 0.6	9.0 ± 0.5

All data were not statistically different between groups.

**Table 2:** The primary outcome.

	NIPPV (143)	NCPAP (143)	Odds Ratio	95% confidence interval	*P*-value
intubation (yes: no; %)	17:126(11.9%)	28:115(19.6%)	2.579	0.862–7.715	0.080
≤32 weeks (73)	11:62(15.1%)	19:54(26.0%)	1.790	0.460–6.969	0.134
32–33 weeks (55)	5:50(9.1%)	8:47(14.5%)	1.536	0.149–15.821	0.549
34–36 weeks (15)	1:14(6.7%)	1:14(6.7%)	0.929	0.803–1.074	1.000
Intubation after surfactant (yes: no; %) (122)	14:108(11.1%)	24:98(19.7%)	2.602	0.784–8.642	0.087
≤32 weeks (56)	7:49(12.5%)	8:48(14.3%)	2.867	0.451–18.212	1.000
32–33 weeks (51)	7:44(13.7%)	16:35(31.4%)	1.788	0.350–9.138	0.049
34–36 weeks (15)	0:15	0:15	—	—	—

**Table 3:** The secondary outcomes.

	NIPPV (143)	NCPAP (143)	Odds Ratio	95% confidence interval	*P*-value
death (yes: no; %)	7:136(4.9%)	12:131(8.4%)	5.040	0.866–29.345	0.302
BPD (yes: no; %)	2:141(1.4%)	2:141(1.4%)	0.986	0.966–1.006	1.000
Frequency of surfactant	1.6 ± 0.9	1.5 ± 0.9	–	(−0.047)−0.187	0.240
Air leak (yes: no; %)	4:139(2.8%)	3:140(2.1%)	0.971	0.944–0.999	1.000
ROP (yes: no; %)	7:136(4.9%)	9:134(6.3%)	2.667	0.286–24.905	0.791
NEC (yes: no; %)	9:134(6.3%)	11:132(7.7%)	3.968	0.717–21.959	0.804
IVH (yes: no; %)	34:109(23.8%)	29:114(20.3%)	1.602	0.649–3.953	0.551
Sepsis (yes: no; %)	39:104(27.3%)	44:99(30.8%)	1.616	0.745–3.507	0.583
PDA (yes: no; %)	13:130(9.1%)	20:123(14.0%)	1.131	0.231–5.529	0.265

bronchopulmonary dysplasia (BPD), patent ductus arteriosus (PDA), retinopathy of prematurity (ROP), necrotizing enterocolitis (NEC), intraventricular hemorrhage (IVH).
